# Effects of open-label placebos on test performance and psychological well-being in healthy medical students: a randomized controlled trial

**DOI:** 10.1038/s41598-021-81502-2

**Published:** 2021-01-22

**Authors:** Julian Kleine-Borgmann, Katharina Schmidt, Marieke Billinger, Katarina Forkmann, Katja Wiech, Ulrike Bingel

**Affiliations:** 1 Department of Neurology, University Hospital Essen, Hufelandstraße 55, 45147 Essen, Germany; 2 Wellcome Centre for Integrative Neuroimaging (WIN), Nuffield Department of Clinical Neurosciences, University of Oxford, Level 6, West Wing, John Radcliffe Hospital, Oxford, OX3 9DU UK

**Keywords:** Cognitive neuroscience, Fatigue, Stress and resilience, Human behaviour

## Abstract

Psychological distress is prevalent in students and can predispose to psychiatric disorders. Recent findings indicate that distress might be linked to impaired cognitive performance in students. Experimental findings in healthy participants suggest that placebo interventions can improve cognition. However, whether non-deceptive (i.e., open-label, OLP) placebos can enhance cognitive function and emotional well-being is unclear. Using a randomized-controlled design we demonstrate a positive impact of OLP on subjective well-being (i.e., stress, fatigue, and confusion) after a 21-day OLP application in healthy students during midterm exams. OLP did not improve test performance, but, within the OLP group, test performance was positively correlated with measures of general belief in the benefit of medication. These results show that OLP can counteract negative effects of acute stress on psychological well-being and might improve cognitive performance if supported by positive treatment expectations. Additionally, our findings in healthy volunteers warrant further investigation in exploring the potential of OLP in reducing stress-related psychological effects in patients. The trial was preregistered at the German Clinical Trials Register on December 20, 2017 (DRKS00013557).

## Introduction

Psychological distress describes a compound of negative feelings, such as emotional suffering, stress, anxiety, and depressed mood. Its prevalence among university students from different faculties in Europe, Australia, the United States and Canada ranges from 20 to 80%^1–4^. Moreover, in the United States and Australia, 15–20% of students meet the criteria for a diagnosis of mental illness^1,5^. It has even been suggested that in the medical student population, psychological distress involving negative mood, anxiety, and stress may predispose to severe psychiatric disorders including substance abuse, major depression, and even suicide^6^.

Levels of psychological distress are particularly high around exam time^7^ and may negatively affect exam performance^8^. For instance, Vedhara et al.^9^ report an exam-associated increase of self-reported stress and a stress-related modulation of cognitive functions (i.e., attention and short-term memory) in students.

While additional research is needed to identify personal and program strategies to promote well-being and prevent cognitive impairment in acutely stressed students^6^, there is, however, cumulating evidence suggesting that positive expectations can have beneficial effects on cognitive performance and emotional well-being in healthy volunteers. The effects of a verbal or contextual manipulation of treatment expectations are referred to as placebo effects^10–12^. Until recently, placebo effects have often been reported after deceptive administration, i.e., individuals being unaware of being treated with a placebo. For instance, Foroughi et al.^13^ investigated the impact of experimentally induced positive expectation on fluid intelligence. In their study, two experimental groups received an identical cognitive training session and fluid intelligence tests were performed before and afterwards. The positive expectation group self-selected the training session responding to an advertisement that suggested cognitive improvement. In contrast, the control group was recruited by a neutral and generic advertisement. Students in the positive expectation group showed improvements that equate to a 5- to 10-point increase on a standard IQ test after the single session, pointing to the impact of expectation on cognitive performance. This is in line with an experimental study by Sinke et al.^14^ reporting positive and negative effects of experimentally modified expectations linked to phasic pain stimuli on short-term memory.

However, deceptive or hidden applications of placebo treatments have raised ethical and legal concerns^15^. To circumvent these challenges, open-label placebo (OLP) interventions administer placebos in an unconcealed fashion with the individual’s awareness and consent. Encouraging pioneering studies suggest beneficial effects of OLP in different clinical conditions, e. g., cancer-related fatigue^16^, chronic back pain^17,18^, allergic rhinitis^19^, attention deficit hyperactivity disorder^20^ or irritable bowel syndrome^21^. A first meta-analysis of these initial studies confirms these effects^22^. However, only few studies investigated non-deceptive (i.e., open-label) placebos in healthy adults. In one trial, both deceptive and non-deceptive (i.e., OLP) placebo interventions equally prevented sensitization of experimentally induced thermal pain^23^, while in another trial using a comparable paradigm only deceptive but not OLP reduced subjective pain ratings^24^. Schneider et al.^25^ investigated an OLP infusion followed by an intracutaneous electrical pain stimulation. Compared to a no-treatment control group, the OLP group showed significantly reduced pain ratings and smaller regions of hyperalgesia and allodynia. A recent trial involving healthy volunteers reported a positive influence of an OLP nasal spray on emotional distress and identified a potential neurobiological correlate for the OLP-induced change^26^. Moreover, Schaefer et al.^27^ found OLP-associated improvements of test anxiety and self-management abilities in a trial enrolling N = 58 students facing an university exam.

In this randomized controlled trial, we investigate the impact of a 3-week open-label placebo treatment on cognitive performance and indicators of subjective well-being including stress, mood, somatization, and depression in healthy medical students before and immediately prior to their midterm medical exams. As performance tests such as exams have been shown to increase perceived stress, mood disturbances, and somatization^28–30^, which can in turn negatively affect test performance^9^, our study design with exam scores as primary outcome measure offers a proof-of-concept investigation into OLP effectiveness in healthy, acutely stressed participants. We hypothesize that (i) OLP improve test performance in healthy medical students, (ii) OLP reduce exam-associated impairment of psychological well-being (i.e., stress, negative mood, somatization, and depression), and (iii) that both effects are linked to positive treatment expectations.

## Methods

### Recruitment and ethics statement

The present study was conducted in accordance with the declaration of Helsinki and approved by the ethics committee of the University Hospital Essen (17-7553-BO). Healthy, young medical students (age: 18–40 years) were recruited by advertisements which were regionally posted at the medical faculty campus of the University Duisburg-Essen, Germany. The advertisement contained information about recent findings suggesting a positive effect of OLP on chronic pain and depression and focused on the primary outcome of the study (i.e., potential cognitive enhancement; see below for details) but also mentioned parameters of psychological well-being which are considered secondary outcomes in this study (for exact wording of the advertisement see supplement). Students aged 18 years or older who had successfully registered for the exam were eligible to take part. Inclusion criteria comprised age ≥ 18 years, successful registration for the exam, and voluntary informed consent. Exclusion criteria comprised self-reported history of severe medical conditions (in particular, diagnosed psychiatric disorders, regular use of psychotropic substances (including alcohol, cannabinoids, cocaine, amphetamines) in the last 4 weeks, known allergy or intolerance to any of the placebo ingredients, current participation in other research studies, and pregnancy. Participants received monetary compensation for their participation and provided written informed consent.

### Participants

**Figure 1:**
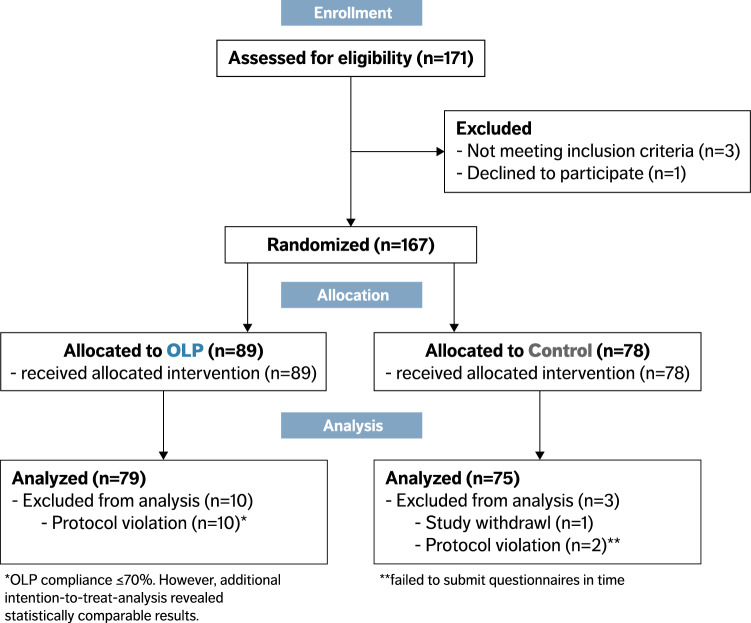
CONSORT 2010 flow diagram. The diagram shows the flow of eligible, randomized, allocated and analyzed participants. Reasons for exclusions are either given in the figure or in “Participants” section. OLP, Open-label placebo.

Of the 167 participants who were enrolled, 166 completed the trial. One participant withdrew due to personal reasons, 12 participants had to be excluded: 2 participants did not submit their data in time, another 10 participants in the OLP group were excluded due to insufficient compliance (for details see Table S1). Our final analyses therefore included 154 participants (104 females [68%], mean age = 24 ± 2.8 years; OLP group = 79 [51.3%], control group = 75 [48.7%]). Self-reported compliance was 94.0 ± 8.1% in the OLP group, indicating high adherence to the treatment schedule. There was no significant group difference in any of the outcome measures at baseline (all *p* > 0.05; for details, see Table 1). See Fig. 1 for flow of participants.

**Table 1:** Baseline characteristics of control and OLP group.

	All (N = 154)	Control (N = 75)	OLP (N = 79)
Female (%)	104 (68)	50 (67)	54 (68)
Age (years)	24.02 ± 2.78	24.08 ± 2.74	23.97 ± 2.83
Body-Mass-Index (kg/m^2^)	22.15 ± 2.71	21.89 ± 2.49	22.39 ± 2.91
Belief about medicines (BMQ-General, Benefits subscale score)	16.25 ± 1.79	16.30 ± 1.87	16.20 ± 1.72
Treatment credibility (CEQ, Credibility subscale score)	0.04 ± 2.59	0.19 ± 2.61	− 0.11 ± 2.57
Treatment expectancy (CEQ, Expectancy subscale score)	0.01 ± 2.37	0.15 ± 2.31	− 0.14 ± 2.42
Trait anxiety (STAI, Trait score)	38.55 ± 8.94	39.13 ± 9.27	38.00 ± 8.64
Depression (CES-D score)	8.06 ± 6.95	9.00 ± 7.52	7.18 ± 6.28
Caffeine consumption (g/day)	4.43 ± 3.54	4.51 ± 3.93	4.35 ± 3.16

Listed are means ± standard deviation.

Please note that the CEQ considers z-transformed values. Thus, a high expectation is represented by positive values, while a low expectation is represented by negative values.

BMQ, Beliefs about Medicines Questionnaire; CEQ, Credibility and Expectancy Questionnaire; STAI, State-Trait-Anxiety Inventory; CES-D, Center for Epidemiologic Studies-Depression Scale.

### Registration

The trial was preregistered at the German Clinical Trials Register on December 20, 2017 (DRKS00013557). Due to legal requirements and in contrast to the originally intended state-run exam, a comparable, standardized, regional exam was used for the test performance assessment (see below). An adapted sample size calculation revealed N = 155 participants to reach a power of 0.9 with an alpha level of 0.05 and an effect size of f = 0.2 (i.e., d = 0.4) using the pwr-package for RStudio^31^. Since data on OLP effects on cognitive performance are sparse, we decided for a sample size calculation based on our recent OLP trial investigating pain relief in chronic back pain with an effect size of d = 0.44^18^. To account for a potential dropout rate of 10% we enrolled N = 165 patients.

### Study design

#### Informed consent and randomization

This randomized, controlled trial recruited 167 healthy students. The general informed consent form which was identical for all participants contained neutral, general information about the placebo effect and a short paragraph summarizing the results of recent open-label placebo trials (e.g. positive effects in pain conditions, see supplement for exact wording). Following the baseline assessment, participants were randomly assigned by a blinded investigator to one of two groups using a sampling algorithm implemented in RStudio (RStudio Version 1.1.463, RStudio, Inc., Boston, MA, USA). While the first group (i.e., open-label placebo, OLP group) received a 21-day OLP (for details see below), the second group (i.e., control group) received no intervention.

#### Open-label placebos

The study was performed single-blinded (blinded investigator) to control for a possible investigator bias. Following baseline assessment, participants in the OLP group received a small cardboard box containing a labeled dispenser with 45 placebo pills (Zeebo, Zeebo Effect, LLC, South Burlington, Vermont, USA) and a note which emphasized that the included pills contained no active ingredient (for full wording see supplement) from the blinded investigator (M.B.) with the verbal instruction to open it at home. The control group received the same box which, however, only contained a note stating that the participant had been assigned to the control group and that no further action was required. Boxes were matched for weight and sound upon shaking to ensure blinding of the investigator who was instructed to avoid any communication about the content and characteristics of the boxes during distribution. Like in previous OLP-trials^17,18,21^, participants in the OLP group were asked to take one placebo pill twice daily for a 21-day application period prior to the exam date. For each participant, placebo intake ended on the exact day of the exam. Self-reported adherence to the treatment schedule was rated on a 101-point computed Visual Analogue Scale (VAS) ranging from 0 to 100 (“How regularly—according to the instructions (2 capsules/day)—have you taken the placebo capsules?” with anchors “not at all”—“as mandated”).

#### Testing schedule

**Figure 2:**
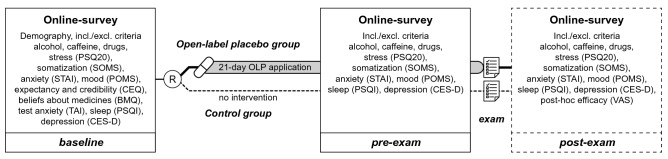
Study design and assessments. The design comprised three online survey assessments: First, prior to randomization [R] (baseline), second, 3 days prior to the exam (pre-exam) and third, 60 days after the exam (post-exam). The latter additionally included the exam score, which was measured as percentage of correct answers in a standardized written midterm medical exam and served as primary outcome. Secondary, i.e. psychometric, outcomes were assessed by standardized questionnaires (PSQ20, Perceived Stress Questionnaire; SOMS, Screening for Somatoform Disorders; STAI, State-Trait-Anxiety Inventory; POMS, Profile of Mood States; CEQ, Credibility and Expectancy Questionnaire; BMQ, Beliefs about Medicines Questionnaire; TAI, Test-Anxiety Inventory; PSQI, Pittsburgh Sleep Quality Index; CES-D, Center for Epidemiologic Studies-Depression Scale; VAS, Visual Analogue Scale) via an online survey. [OLP, Open-label placebos].

The testing schedule comprised three time points—baseline, pre-exam and post-exam. All outcomes were assessed via a self-administered online survey system (LimeSurvey, LimeSurvey GmbH, Hamburg, Germany). The baseline assessment took place on the same day that participants were enrolled in the study at the facilities of the medical faculty of the University Duisburg-Essen and comprised obtaining information about demographic characteristics, alcohol, nicotine and caffeine consumption and drug use. Furthermore, participants completed the following questionnaires: PSQ20, POMS, SOMS, STAI-T, CES-D, CEQ, BMQ and TAI (for details see below). Pre-exam measures were scheduled three days prior to the exam. They included information about alcohol, caffeine and drug consumption, as well as completion of PSQ20, POMS, SOMS, STAI-S, and CES-D. The post-exam assessment was scheduled 60 days after the exam and comprised post-hoc treatment efficacy ratings in addition to the assessments performed at pre-exam timepoint and intended to request the documented individual exam performance. Furthermore, it included open questions to obtain individual feedback about the OLP application as well as to retrospectively assess adverse effects. For an overview of the testing schedule, see Fig. 2. All participants were automatically contacted via a standardized email three days before (pre-exam) and 60 days after exam (post-exam) and asked to complete the online assessments (for detailed time frames see Table 2). Note that due to logistical reasons, subjective outcome measures were assessed three days before the exam. OLP intake was continued until the day of the examination.

**Table 2:** Timing details of the scheduled timepoints.

Timeframe (in days)	All (N = 154)	Control group (N = 75)	OLP group (N = 79)
Baseline to exam	53.39 ± 14.82	53.71 ± 14.98	53.09 ± 14.76
Pre-exam to exam	2.78 ± 1.34	2.75 ± 1.33	2.81 ± 1.36
Exam to post-exam	61.13 ± 7.93	60.83 ± 8.05	61.41 ± 7.87

Listed are the means and standard deviations of time periods between the assessments and the exam in days.

### Outcome measures

In addition to the primary and secondary outcomes described below, the survey included standardized questionnaires to obtain demographic information and assess average alcohol and drug consumption within a 7-day period as well as inclusion/exclusion criteria. For details of the assessment schedule see Fig. 2.

#### Primary outcome

Primary outcome was the exam score of a standardized written midterm medical exam which was calculated as percentage of correct answers. Students are required to take part in this bi-annual standardized exam with a multiple-choice response format. For all students, the exam was completed on 2 days and included 100–200 questions depending on the academic year (OLP group: 176 ± 28, control group: 175 ± 31 questions).

#### Secondary outcomes

Secondary outcomes included the change in scores assessing stress, mood, somatization, depression and anxiety from baseline to pre-exam. Stress was assessed using the validated Perceived Stress Questionnaire (PSQ20)^32^ which provides a total score and scores of the subscales ‘worries’, ‘tension’, ‘joy’ and ‘demands’. Current state of mood was rated using the Profile of Mood States questionnaire (POMS)^33^ which is comprised of seven subscales (total mood disturbance, tension-anxiety, depression, anger-hostility, vigor-activity, fatigue, and confusion-bewilderment). Somatization was assessed with the Screening of Somatoform Disorders (SOMS) questionnaire^34^, which gives both a symptom and an intensity score. Participants also completed the Center for Epidemiologic Studies-Depression Scale (CES-D)^35^ for symptoms of depression and the State-Trait-Anxiety Inventory (trait and state version: baseline: STAI-T, pre-exam: STAI-S)^36^ for anxiety.

#### Explorative outcomes

Explorative outcomes included change in sleep quality and self-reported caffeine consumption in a 7-day period [mg/day]. Both explorative outcomes were assessed at baseline and pre-exam. The average sleep quality over the past 4 weeks was assessed using the Pittsburgh Sleep Quality Index (PSQI)^37^. Participants were asked to report caffeine consumption within the last seven days in mg/day using an in-house questionnaire. Additional positive or negative (adverse) effects were recorded by an open response format (“Have you noticed positive (negative) effects of the open-label placebo application? If so, which?”). For subgroup analyses, which are not reported in this manuscript, we included further assessments of personality factors, activity and sports, learning strategies and locus of control by standardized questionnaires.

#### Covariates

Covariates were assessed at baseline and included treatment expectations and rationale credibility of the open-label placebo treatment (Credibility and Expectancy Questionnaire, CEQ^38^, general beliefs about medicines (Beliefs About Medicines-General-12 questionnaire, BMQ^39^, an established instrument to assess perceptions and expectations about medications^40^ including a four-item General-Benefit scale assessing individuals’ belief about potential benefits of medicines^41^; and test anxiety (Test Anxiety Inventory, TAI^42^). The OLP group rated OLP treatment efficacy (“How do you think the placebo application influenced your exam performance?”) upon completion of the treatment using a 101-point computerized VAS with numerical anchor points − 50 (“very negatively”; left anchor) and 50 (“very positively”; right anchor).

### Statistical analysis

Statistical analyses were performed using RStudio (RStudio, version 1.2.5033, RStudio Team, RStudio: Integrated Development for R. RStudio, Inc., Boston, MA). Participants were excluded from data analysis if (i) they failed to submit their data in time (≤ 1 h prior to exam) or (ii) self-reported adherence to the treatment schedule (i.e., compliance) was insufficient (i.e., ≤ 70%, as recommended for the analysis of clinical trials;^43^. Intervention effects on the primary outcome were tested using a general linear model to compare exam scores between groups (open-label placebo, no treatment; between-subject factor). Secondary outcomes were tested for differences between groups (open-label placebo, no treatment; between-subject factor) in pre-post-treatment changes (baseline, pre-exam; within-subject factor) using general linear mixed models. Separate models were calculated for each outcome measure. In line with our hypotheses regarding the impact of expectations on the placebo effect^44^, the CEQ expectancy and BMQ benefit subscales were included as covariates in the models. For results, non-standardized (ß, reported in the “Results” section) and standardized (B; see supplement) estimates of means differences ± standard errors (SE) are provided. Statistical testing was performed at alpha < 0.05. Descriptive results are provided as means ± standard deviation (SD).

### Statement of ethics

The present study was conducted in accordance with the declaration of Helsinki and approved by the ethics committee of the University Hospital Essen (17-7553-BO). All participants gave voluntary written informed consent for participation and publication.

## Results

Of the 167 participants enrolled, 166 completed the trial. Due to exclusion of 12 participants (for details see “Methods” section and Table S1) our final analyses included 154 participants (104 females [68%], mean age = 24 ± 2.8 years; OLP group = 79 [51.3%], control group = 75 [48.7%]). Statistical analyses were performed using a general linear model to compare exam scores between groups (between-subject factor). Secondary outcomes were tested for differences between groups in pre-post-treatment changes (within-subject factor) using general linear mixed models. Please note that an additionally performed intention-to-treat analysis including all participants revealed comparable results (data not shown).

### Main outcome: exam score

**Figure 3:**
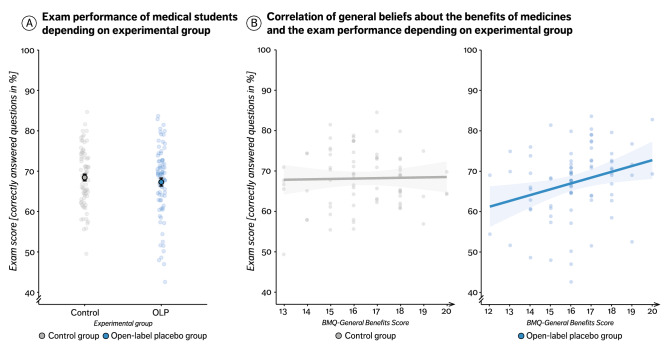
A and B. Impact of open-label placebos on the test performance. (**A**) Exam scores (percentage of correct answered questions out of all questions answered) separately for both groups. Displayed are mean values (single, black surrounded dots) ± standard error of the mean, and single subject scores (faded blue or grey dots). (**B**) Correlation between the BMQ benefit score and the exam score in the OLP group (r = 0.28, t(77) = 2.58, *p* = .011, right) and control group (r =  − 0.03, t(73) =  − 0.30, *p* = .766, left). Displayed are single subject scores (faded blue or grey dots) and regression lines.

The comparison of exam scores between groups did not yield a significant result. However, inclusion of the general beliefs about medicines questionnaire (BMQ) ‘Benefits’ subscale as a covariate revealed a significant interaction between group and treatment expectation (group × BMQ 'Benefits’ score; ∆OLP-control group = 1.57 ± 0.71%, t(153) = 2.21, *p* = 0.029, d = 0.18), indicating that a generally higher expectation to benefit from medication was linked to better test performance in OLP but not the control group. Similarly, adding the participant’s expectation of the OLP treatment (expectancy subscale score of the Credibility and Expectancy Questionnaire, CEQ) as a covariate to the analysis showed a trend towards a significant interaction effect (group × CEQ expectation score, OLP compared to control, 0.96 ± 0.55%, t(153) = 1.94, *p* = 0.074, d = 0.15). Both results suggest that beliefs and expectation might modulate OLP effects. Figure 3 shows exam scores and the correlation with the BMQ ‘Benefits’ score separately for both groups.

### Secondary and explorative outcomes: well-being

**Figure 4:**
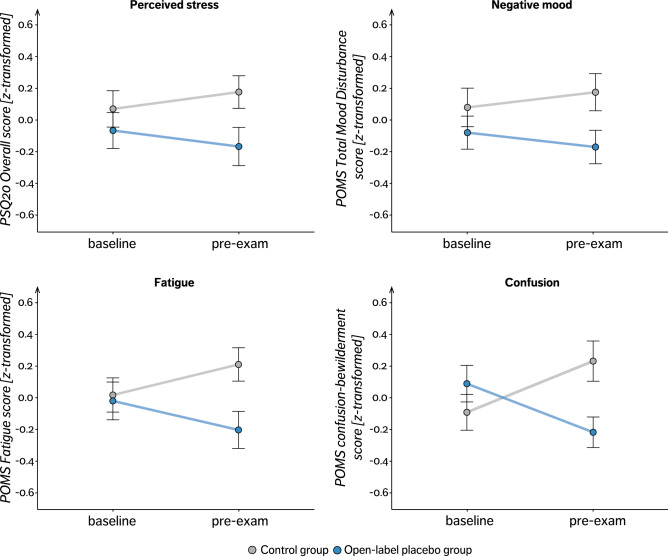
Secondary outcome analyses. Displayed are changes in PSQ20 Overall Stress Score and POMS subscales separately for both groups as normalized (z-transformed) mean values (single, black surrounded dots) ± standard error of the mean. PSQ20, Perceived Stress Questionnaire; POMS, Profile of Mood States. Please note that for better comparability of the subscale scores a z-transformation was performed with positive values representing a positive, and negative values representing a negative deviation from the sample’s mean value. For non-standardized charts see Fig. S1.

Overall, our total study sample showed an increase in all secondary (i.e., stress, negative mood, somatization, anxiety, depression) and explorative outcomes (i.e., sleep quality impairment, caffeine consumption) from baseline to pre-exam assessment. Participants in the OLP group showed a significantly smaller increase in overall stress scores (Perceived Stress Questionnaire, PSQ20) from baseline to pre-exam in comparison to control group participants as indicated by a group × time interaction (OLP compared to control, − 4.84 ± 2.44, t(153) =  − 1.98, *p* = 0.049, d = −0.32). The PSQ20 subscale ‘Joy’ showed a trend towards an interaction (group × time, OLP compared to control, 5.00 ± 2.96, t(153) = 1.69, *p* = 0.093, d = 0.21), indicating a smaller decrease in joy in the OLP compared to the control group. Other PSQ20 subscale analyses did not reveal any significant effects. The Profile of Mood States (POMS) ‘Total Mood Disturbance’ score showed a trend decrease from baseline to pre-exam in the OLP group but an increase in the control group (group × time, OLP compared to control, − 7.41 ± 4.02, t(153) =  − 1.84, *p* = 0.068, d = − 0.29). Further subscale analyses for the POMS revealed a significantly smaller increase in fatigue (group × time, OLP compared to control, − 2.40 ± 0.83, t(153) =  − 2.90, *p* = 0.004, d = − 0.47) and in confusion scores (group × time, OLP compared to control, − 2.12 ± 0.55, t(153) =  − 3.83, *p* < 0.001, d = − 0.62; see Fig. 4) in the OLP than the control group. The analyses of the subscales ‘Anger’ and ‘Vigor/Activity’ revealed no statistically significant group differences. Including either BMQ ‘Benefits’ subscale scores or CEQ expectancy subscale scores did not lead to model improvement. However, focusing on the OLP group only, a correlation analysis revealed a significant negative association between the expectation (BMQ general benefits score) and the PSQ20 tension score, indicating that higher expectation scores were associated with smaller increase in tension (r = − 0.24, t(71) =  − 2.23, *p* = 0.028). Furthermore, we found a trends towards a negative correlation between expectation scores (BMQ general benefits score) and the intensity (r = − 0.20, t(71) =  − 1.82, *p* = 0.072) and number of symptoms (Screening for Somatoform Disorders (SOMS) intensity and symptom score, r = − 0.18, t(71) =  − 1.67, *p* = 0.098) which is indicative of a smaller increase in somatization with higher expectation scores. There were no statistically significant group differences with respect to depression (Center for Epidemiologic Studies-Depression Scale, CES-D), anxiety (State-Trait-Anxiety Inventory, STAI-S) and sleep quality (Pittsburgh Sleep Quality Index, PSQI), test anxiety (Test-Anxiety Inventory, TAI) as well as in caffeine consumption (all *p* > 0.05, results are listed in Table S3 and depicted in Fig. S2). As part of the open format feedback, one participant in the OLP group reported flatulence as a treatment side effect. Five students provided negative feedback: four participants reported “worries about forgetting to take the tablets regularly” and one highlighted “the inconvenience of the capsule intake twice daily”. Eleven students gave positive feedback including “that the OLP helped them structure their daily routine”, “increased their motivation”, had an “overall encouraging effect”.

## Discussion

This prospective, randomized, controlled trial in 154 medical students shows that a three-week OLP treatment can significantly reduce exam-related distress, as indexed by a reduction in perceived stress, negative mood, fatigue and confusion. Although OLP treatment did not lead to higher test scores across the sample, it improved test performance in those with a strong belief in the benefit of medication (BMQ general benefits score). These findings obtained in healthy students may generalize to other healthy individuals undergoing periods of increased demand for various reasons and may motivate future investigations in patients suffering from distress and cognitive impairment.

### OLP counters exam-related negative effects on well-being

Exams are known to induce stress which is reflected in subjective as well as objective measures^28–30^ and can in turn compromise task performance^9^. In our cohort of medical students, preparation for the exam was linked to an increase in stress, negative mood, somatization, depression, anxiety, compromised quality of sleep quality, and self-reported caffeine consumption prior to the exam. OLP were able to prevent the increase in perceived stress, fatigue, and confusion, indicating a positive effect on parameters of well-being and reduction of exam-related distress in healthy individuals (Fig. 4). The OLP group also showed less increase in all other outcome variables including mood impairment, anxiety, somatization, sleep quality impairment, and caffeine consumption although these changes did not reach statistical significance.

Positive effects of OLP on performance-related distress had previously only been shown in studies with considerably smaller sample sizes. Following a very early observation of global improvement and symptom relief in N = 14 “neurotic patients” by Park and Covi^45^, beneficial effects of open-label placebos were first systematically investigated in patients suffering from attention deficit hyperactivity disorder^20^. Since then, other studies have reported significant effects of OLP on mental health outcome related parameters such as cancer-related fatigue or QoL in IBS patients, and perceived stress in patients with chronic low back pain^16–18,21^. The present study extends these findings by showing a moderate to large effect of OLP on parameters of well-being in a large cohort of 154 healthy volunteers and demonstrates that placebo effects can be harnessed in a real-life scenario without deception. These findings are in line with a recent study demonstrating beneficial effects of OLP on experimentally induced psych distress and affect related neurophysiology in a large experimental study^26^. Although there is strong evidence that exam situations impair well-being^28–30^, it should be pointed out that the steeper increase of distress in the control group could also be driven by participants in this group who felt disappointed that they did not receive the placebo. While clinical trials offer ways to reduce the potential influence of this factor (e.g., by offering OLP administration after trial participation^17,18^), our study design was naturally limited to a one-time treatment phase (i.e., the exam situation).

### No main effect of OLP on task performance

Contrary to our hypothesis, OLP treatment had no significant effect on cognitive test performance across the group (Fig. 3A). To our knowledge, OLP effects on cognitive/test performance have not been investigated so far. A recent study^46^ (N = 58) showed that OLP reduced test anxiety but did not include test performance as an outcome variable. Previous studies using deceptive placebo treatments or manipulations of expectancy on objective outcome parameters of cognitive performance had yielded very mixed results^47,48^, suggesting that placebo effects on cognitive performance might be complex and dependent on a variety of factors. Our results suggest that the same applies to open-label application of placebos. Interestingly, differential effects on subjective and objective outcome parameters has also been observed in traditional hidden placebo trials^15,49^ as well as in OLP treatment of chronic back pain patients^18^. Winkler and Hermann^48^ for instance have shown that a placebo, which was experimentally linked to either a positive (i.e., placebo-nootropic) or negative expectation (i.e., placebo-antihistamine agent), improved perceived but not actual cognitive performance in a standardized test.

Although OLP showed no effect on exam scores across the sample, it did modulate performance in a subset of participants. More specifically, OLP treatment led to better test performance in those who generally believe in the benefit of medication (BMQ general benefits score) and might have even worsen test performance in those who did not (lower BMQ general benefit scores). Inter-individual variability in placebo responses is a well-known phenomenon^50^ and differences in treatment expectations are seen as a key reason of this heterogeneity. Whether expectations are as important for the efficacy of OLP as for hidden placebos is currently under debate^51^. Our findings indicate that rather than specific treatment expectations, a more generic belief in the benefit of medication differentiated between individuals as participants with a strong general belief in the value of medication showed improved performance under OLP. This finding seems surprising at first, given that participants were aware that the pills they received contained no active ingredient and did therefore not conform with the common definition of ‘medication'. It seems reasonable to assume that somebody with a strong belief in the benefits of conventional medication (as assessed in the BMQ) should therefore be less likely to believe in and benefit from OLP. However, our data rather suggest that our participants considered OLP a treatment strategy that is given similar credit as conventional medication.

### OLP are well tolerated

In contrast to many active treatments such as pharmacological cognitive enhancers which are increasingly consumed by students^52^ and are often accompanied by adverse effects^53,54^, OLP were safe and well tolerated in our study. Only one participant reported adverse effects (flatulence) and five students provided negative feedback (i.e., worries about forgetting to take the OLP regularly). In contrast, eleven of seventeen students gave positive feedback including that the OLP helped them to structure their daily routine, led to higher motivation, and had an encouraging effect. Moreover, self-reported OLP compliance (adjusted sample: 94.0%, full sample: 88.7%) was higher than commonly reported in clinical trials (43 to 78%)^43^.

### Implication for future studies

Our findings obtained in healthy students may have several implications for future studies. The mechanisms underlying OLP are still widely unknown. Although mechanisms such as expectation, cognitive prediction processes^51^ and natural fluctuation, have been suggested to contribute, further research is needed to explore the (relative) significance of these and further factors as well as their underlying neurobiological mechanisms. Future trials need to test whether our results can be replicated in the general population, whether OLP treatment can have long-term effects, and which factors moderate and predict beneficial effects of OLP on emotional well-being. Stress, fatigue and confusion, which improved under OLP treatment, are not specific to an exam situation, which was chosen here because it constitutes a highly controlled setting with a simple outcome measure in healthy volunteers. However, they are also common in chronic mental and somatic diseases such as depression, multiple sclerosis or chronic pain where they contribute significantly to disease-related disability^55–57^. Although OLPs had no significant effect on exam performance across our sample of healthy students, it remains to be explored whether it could be used to improve cognitive functioning in other populations.

### Limitations

Our results must be interpreted in the light of the following limitations. First, like in other studies in which participants respond to a public call for volunteers, our trial was susceptible to a self-selection bias and might have particularly attracted those with an interest in OLP treatment and a positive attitude towards this kind of treatment. Second, as discussed above, exam performance depends on a multitude of factors such as the students’ preparation, motivation and concentration which were not controlled for in our study. However, our approach complements highly controlled experimental trials as it offers a high degree of ecological validity which facilitates the translation of key findings into clinical practice. Third, exam-related distress is limited in time and the effects we found might therefore not necessarily translate to prolonged stress. Finally, like in other OLP trials^17,18,21^, the fact that participants were not blinded to the treatment might have introduced a reporting bias. Open communication and administration of the placebo is an integral part of the OLP rationale, which renders controlling for this type of bias difficult. Designing clinical OLP trials is challenging due to the lack of rigorous control groups, a potential bias of experimenters, and the not yet fully understood relevance of the rationale on OLP efficacy^58,59^. However, this study tried its best to reduce these constraints, i.e., both experimental groups were structurally equivalent, were randomly assigned, received identical information regarding the rationale of the treatment and only differed in the OLP treatment. Further, the quantity and quality of interactions with the study personnel was identical in both groups and the experimenter was blinded to treatment allocation.

## Conclusions

In sum, our study shows that a 3-week OLP treatment can improve well-being in acutely stressed healthy students. While no general effect on test performance was observed, OLP increased cognitive performance in those with a general belief in the benefits of medication. In contrast to currently available treatment options, OLP were safe and well tolerated. Given the prevalence of symptoms of distress and fatigue in the general population and patients suffering from chronic health conditions, OLP might be a useful addition to the therapeutic portfolio, particularly in patients who are open to such intervention, or those with contraindications to current gold standard treatments.

## Supplementary Information

Supplementary Information 1.

## Data Availability

Data will be shared upon reasonable request addressed to Julian.Kleine-Borgmann@uk-essen.de.
