# Supplementary material for: Effects of open-label placebos on test performance and psychological well-being in healthy medical students: a randomized controlled trial
Source: Sci Rep. 2021 Jan 22;11:2130. doi: 10.1038/s41598-021-81502-2 (PMC7822842; doi:10.1038/s41598-021-81502-2)
Supplement: Supplementary file 1 — Supplementary Information 1. [file 41598_2021_81502_MOESM1_ESM.docx]

**SUPPLEMENT TO**

**Effects of open-label placebos on test performance and psychological well-being in healthy medical students: A randomized controlled trial**

Julian Kleine-Borgmann^1^*, Katharina Schmidt^1^, Marieke Billinger^1^, Katarina Forkmann^1^, Katja Wiech^2^, Ulrike Bingel^1^

^1^University Hospital Essen, Department of Neurology, Hufelandstraße 55, 45147 Essen, Germany.

^2^Wellcome Centre for Integrative Neuroimaging (WIN), Nuffield Department of Clinical Neurosciences, University of Oxford, Level 6, West Wing, John Radcliffe Hospital, Oxford OX3 9DU, United Kingdom.

*Corresponding author:

Julian Kleine-Borgmann

University Hospital Essen

Department of Neurology

Hufelandstraße 55

D-45147 Essen

Tel: +49 201 723 – 2446

Email: julian.kleine-borgmann@uk-essen.de

Supplementary Materials:

*Information provided for the participants*

Please note that due to a logistical error, our participant information sheet contained information about the expected effect on well-being, whereas our advertisements for recruitment unfortunately did not. However, participants were informed about the expected effect on psychological well-being as they all received the participant information sheet which was, moreover, handed out after they had seen the advertisements.

*Excerpt of the advertisement*

For a study of the Department of Clinical Neurosciences we are recruiting healthy students who are in preparation for their final exams of the second part of their medical studies. We aim to investigate the impact of an open-label placebo application on cognitive performance. The study includes one appointment on the campus (approx. 1 hour) and two online survey sessions (approx. 30 minutes/session).

*Excerpt of the informed consent explaining the rationale, full wording, translated from German*

Meta-analyses confirm that placebo treatments (inert drug missing an active ingredient) can have significant and clinically relevant effects on various conditions. Astonishingly, in conditions of acute and chronic pain and depression, for example, it has been shown that these positive effects also occur when the patient is aware of being treated with a placebo (so-called open-label placebo treatment (OLP)). The underlying mechanisms of this effect are not yet known. In particular, evolutionary conditioning processes but also psycho-neurobiological mechanisms of increased self-regulation/self-efficacy are discussed. Recent studies show that cognitive functions and well-being can be influenced by the placebo effect, especially in test situations. So far it is unknown whether open-label placebos can also influence cognitive performance and well-being, and which mechanisms are involved. This will be further investigated in this study. The test performance in the written semi-annual examination will be the objective criterion of cognitive performance in this study.

*Information handed out at randomization*

*Allocation to the OLP group*

Dear participant, you have been assigned to the group receiving a placebo application. Enclosed you will find the placebo capsules. Please open the container and discard the small bag that is intended to keep the capsules dry. Please take one capsule in the morning and one capsule in the evening at the beginning of the test period (in total 2 capsules per day) for 21 days. Please find the beginning’s date of the test period labeled on the container. We will also send you an e-mail reminder. Please make sure that you take the capsules regularly. Please store the container at room air and dry atmosphere. Please note the following when contacting us: The group allocation is blinded, so that your contact person does not know your group allocation and should only know it in case of an emergency. Many thanks for your cooperation!

*Allocation to the control group*

Dear participant, you have been assigned to the observation group. You will not receive placebo capsules during this study. Please find the beginning’s date of the test period labeled on the container. We will also send you an e-mail reminder. There is no need for further actions after receiving the e-mail.

##

Fig. S1 – Secondary outcome analyses (non-standardized). Displayed are changes in PSQ20 Overall Stress Score and POMS subscales separately for both groups. PSQ20 = Perceived Stress Questionnaire, POMS = Profile of Mood States. Displayed are mean values (single, black surrounded dots) ± standard error of the mean, and single subject scores (faded blue or grey dots).

##

Figure S2 – Normalized results of the secondary and exploratory outcomes. The figure shows the group-depending change of z-transformed mean values (single, black surrounded dots) ± standard error of the mean from baseline to pre-exam. Although these results are not statistically significant, they descriptively support the hypothesis of an OLP effect on these reported outcomes. POMS = Profile of Mood States; STAI = State-Trait-Anxiety-Inventory; PSQ20 = Perceived Stress Questionnaire; SOMS = Screening for Somatoform Disorders; PSQI = Pittsburgh Sleep Quality Index.

| **ID** | **Reason for exclusion** |
| --- | --- |
| 1012 | 46% OLP-compliance |
| 1028 | 37% OLP-compliance |
| 1035 | 20% OLP-compliance |
| 1050 | 64% OLP-compliance |
| 2032 | 55% OLP-compliance |
| 2036 | 39% OLP-compliance |
| 2045 | 58% OLP-compliance |
| 2054 | 59% OLP-compliance |
| 3001 | 24% OLP-compliance |
| 3008 | 68% OLP-compliance |
| 2001 | Withdrawal from exam participation |
| 1021 | Pre-exam submission date after exam date |
| 1010 | Pre-exam submission date after exam date |

**Table S1 Individual reasons for exclusion of participants.** Participants’ identification numbers (ID) and exclusion reasons.

| **Dependent variable: Mean exam score [%]** | | | | | |
| --- | --- | --- | --- | --- | --- |
| **Interactions** | **β ± SD in %** | **B ± SD** | **t-value** | **p-value** | **Cohen’s d** |
| OLP application | -1.12 ± 1.30 | -0.07 ± 0.16 | -0.87 | .388 | -0.07 |
| OLP application × BMQ Benefits | +1.57 ± 0.71 | 0.54 ± 0.09 | 2.21 | .029 | 0.18 |
| OLP application × CEQ expectancy | +0.96 ± 0.55 | 0.21 ± 0.06 | 1.94 | .074 | 0.15 |

**Table S2: Results of the primary outcome.** General linear model considering the control group as reference group. Degrees of freedom (df) = 153. β represent estimated mean differences, B normalized (z-transformed) estimates; SD: standard deviation; BMQ: Beliefs about Medicine Questionnaire; CEQ = Credibility and Expectancy Questionnaire.

| **Interactions (group** × **time)** | **β ± SD in %** | **B ± SD** | **t-value** | **p-value** | **Cohen’s d** |
| --- | --- | --- | --- | --- | --- |
| POMS, Total Mood Disturbance | -7.41 ± 4.02 | -0.12 ± 0.06 | -1.84 | .068 | -.29 |
| POMS, Fatigue | -2.40 ± 0.83 | -0.18 ± 0.06 | -2.90 | .004 | -.47 |
| POMS, Confusion | -2.12 ± 0.55 | -0.26 ± 0.07 | -3.83 | <.001 | -.62 |
| POMS, Anger | -1.00 ± 0.86 | -0.09 ± 0.08 | -1.16 | .247 | -.19 |
| POMS, Depression | -1.38 ± 1.20 | -0.08 ± 0.07 | -1.15 | .251 | -.19 |
| POMS, Vigor/Activity | -0.02 ± 0.97 | -0.01 ± 0.06 | -0.02 | .984 | -.01 |
| PSQ20, Overall | -4.84 ± 2.44 | -0.11 ± 0.05 | -1.98 | .049 | -.32 |
| PSQ20, Joy | +5.00 ± 2.96 | +0.09 ± 0.06 | 1.69 | .093 | .21 |
| PSQ20, Demands | -6.17 ± 3.73 | -0.11 ± 0.07 | -1.65 | .101 | -.27 |
| PSQ20, Worries | -2.30 ± 2.64 | -0.06 ± 0.05 | -1.14 | .258 | -.18 |
| PSQ20, Tension | -5.17 ± 3.20 | -0.10 ± 0.06 | -1.62 | .108 | -.26 |
| SOMS, Intensity | -0.80 ± 1.72 | -0.03 ± 0.06 | -0.47 | .642 | -.08 |
| SOMS, Symptoms | -0.91 ± 0.95 | -0.06 ± 0.06 | -0.96 | .341 | -.16 |
| STAI-S | -1.44 ± 1.47 | -0.06 ± 0.06 | -0.98 | .328 | -.16 |
| CES-D | -0.20 ± 1.05 | -0.01 ± 0.06 | -0.19 | .853 | -.03 |
| PSQI | -0.59 ± 0.46 | -0.09 ± 0.07 | -1.29 | .201 | -.21 |
| Caffeine [mg/d] | -12.23 ± 14.61 | -0.07 ± 0.08 | -0.84 | .404 | -.16 |

**Table S3: Results of the secondary and exploratory outcomes.** Linear mixed model considering the control group as reference group. Degrees of freedom (df) = 153. β represent estimated mean differences, B normalized (z-transformed) estimates; SD = standard deviation, POMS = Profile of Mood States, PSQ20 = Perceived Stress Questionnaire, SOMS = Screening for Somatoform Disorders, STAI = State-Trait-Anxiety Inventory, CES-D = Center for Epidemiologic Studies-Depression Scale, PSQI = Pittsburgh Sleep Quality Index.

| **Outcome** | **Control (N=75)** | | | **OLP (N=79)** | | |
| --- | --- | --- | --- | --- | --- | --- |
|  | ***baseline*** | ***pre-exam*** | ***delta*** | ***baseline*** | ***pre-exam*** | ***delta*** |
| ***PSQ20*** | | | | | | |
| Overall | 35.02±15.98 | 51.76±18.24 | 16.73±14.66 | 32.83±16.18 | 44.73±21.90 | 11.90±15.57 |
| Demands | 41.78±22.81 | 58.67±21.25 | 16.89±24.07 | 41.69±20.57 | 52.41±26.55 | 10.72±22.28 |
| Tension | 35.11±18.87 | 55.47±21.62 | 20.36±19.63 | 31.05±19.68 | 46.24±25.58 | 15.19±20.03 |
| Worries | 25.78±19.65 | 38.31±23.04 | 12.53±15.84 | 22.53±17.32 | 32.07±24.04 | 9.54±16.83 |
| Joy | 62.58±17.26 | 45.42±22.35 | -17.16±19.64 | 63.97±21.58 | 51.81±22.79 | -12.15±17.04 |
| ***POMS*** | | | | | | |
| Total mood disturbance | 5.57±18.71 | 34.84±30.01 | 29.27±26.27 | 2.75±16.47 | 24.61±27.86 | 21.86±23.64 |
| Fatigue | 5.75±4.11 | 12.32±5.65 | 6.57±5.14 | 5.58±4.66 | 9.76±6.44 | 4.18±5.13 |
| Anger-hostility | 3.15±3.60 | 6.79±6.28 | 3.64±5.76 | 2.71±3.18 | 5.35±4.98 | 2.65±4.83 |
| Vigor-activity | 16.93±5.75 | 12.57±6.56 | -4.36±5.54 | 18.33±6.41 | 13.95±7.04 | -4.38±6.38 |
| Confusion-bewilderment | 3.79±2.46 | 7.83±4.06 | 4.04±4.12 | 4.24±2.57 | 6.16±3.16 | 1.92±2.61 |
| Depression | 3.77±6.01 | 9.59±8.93 | 5.81±7.71 | 3.05±4.19 | 7.48±7.56 | 4.43±7.19 |
| ***SOMS*** | | | | | | |
| Intensity | 10.37±10.30 | 16.95±13.10 | 6.57±10.78 | 10.34±9.86 | 16.11±14.26 | 5.77±10.54 |
| Symptoms | 6.85±5.56 | 11.09±7.62 | 4.24±6.45 | 6.89±5.52 | 10.23±7.74 | 3.33±5.35 |
| ***Other assessments*** | | | | | | |
| ***PSQI*** | 5.99±3.04 | 6.77±3.25 | 0.88±3.08 | 5.36±2.49 | 5.64±2.79 | 0.22±2.41 |
| ***STAI-S*** | 36.16±7.79 | 47.87±11.05 | 11.71±8.73 | 35.10±8.64 | 45.37±11.10 | 10.27±9.46 |
| ***CES-D*** | 9.00±7.52 | 14.11±7.16 | 5.11±7.00 | 7.18±6.28 | 12.09±6.47 | 4.91±6.07 |
| ***Caffeine*** | 68.50±68.38 | 113.47±88.2 | 44.97±82.53 | 66.52±56.56 | 98.61±94.04 | 32.09±83.67 |

**Table S4: Summary of outcomes assessed at baseline and pre-exam.** Listed are mean values ± standard deviation of raw data of the assessed outcomes at baseline and pre-exam as well as the difference of pre-exam and baseline scores (deltas); SD = standard deviation, PSQ20 = Perceived Stress Questionnaire, POMS = Profile of Mood States, SOMS = Screening for Somatoform Disorders, PSQI = Pittsburgh Sleep Quality Index, STAI = State-Trait-Anxiety Inventory, CES-D = Center for Epidemiologic Studies-Depression Scale.
